# The use of sequential mark-release-recapture experiments to estimate population size, survival and dispersal of male mosquitoes of the  *Anopheles gambiae* complex in Bana, a west African humid savannah village

**DOI:** 10.1186/s13071-017-2310-6

**Published:** 2017-08-07

**Authors:** Patric Stephane Epopa, Abdoul Azize Millogo, Catherine Matilda Collins, Ace North, Frederic Tripet, Mark Quentin Benedict, Abdoulaye Diabate

**Affiliations:** 1 Institut de Recherche en Sciences de la Santé / Centre Muraz, Bobo-Dioulasso, Burkina Faso; 2 Institut des Sciences des Sociétés, Ouagadougou, Burkina Faso; 3 Centre for Environmental Policy, Imperial College London, London, UK; 4 Department of Zoology, University of Oxford, Oxford, UK; 5 Centre for Applied Entomology and Parasitology, School of Life Sciences, Keele University, Keele, Staffordshire UK; 6 Centers for Disease Control and Prevention (CDC), Atlanta, USA

**Keywords:** Mark-release-recapture, *Anopheles coluzzii*, Population size, Survival, Dispersal, Male mosquitoes, Genetic control

## Abstract

**Background:**

Vector control is a major component of the malaria control strategy. The increasing spread of insecticide resistance has encouraged the development of new tools such as genetic control which use releases of modified male mosquitoes. The use of male mosquitoes as part of a control strategy requires an improved understanding of male mosquito biology, including the factors influencing their survival and dispersal, as well as the ability to accurately estimate the size of a target mosquito population. This study was designed to determine the seasonal variation in population size via repeated mark-release-recapture experiments and to estimate the survival and dispersal of male mosquitoes of the *Anopheles gambiae* complex in a small west African village.

**Methods:**

Mark-release-recapture experiments were carried out in Bana Village over two consecutive years, during the wet and the dry seasons. For each experiment, around 5000 (3407–5273) adult male *Anopheles coluzzii* mosquitoes were marked using three different colour dye powders (red, blue and green) and released in three different locations in the village (centre, edge and outside). Mosquitoes were recaptured at sites spread over the village for seven consecutive days following the releases. Three different capture methods were used: clay pots, pyrethroid spray catches and swarm sampling.

**Results:**

Swarm sampling was the most productive method for recapturing male mosquitoes in the field. Population size and survival were estimated by Bayesian analyses of the Fisher-Ford model, revealing an about 10-fold increase in population size estimates between the end of dry season (10,000–50,000) to the wet season (100,000–500,000). There were no detectable seasonal effects on mosquito survival, suggesting that factors other than weather may play an important role. Mosquito dispersal ranged from 40 to 549 m over the seven days of each study and was not influenced by the season, but mainly by the release location, which explained more than 44% of the variance in net dispersal distance.

**Conclusion:**

This study clearly shows that male-based MRR experiments can be used to estimate some parameters of wild male populations such as population size, survival, and dispersal and to estimate the spatial patterns of movement in a given locality.

## Background

In spite of substantial investment over many decades and the accumulation of an important body of knowledge related to malaria, in 2015 there were still more than 400,000 deaths from malaria, 90% of which were in sub-Saharan Africa [1]. Research directed towards malaria control covers a diverse area of expertise such as pathology, epidemiology, immunology, entomology, health economics and sociology. Particular emphasis has typically been put on three main objectives: understanding the biology of the parasites and vectors; improving the understanding of disease epidemiology; and developing efficient control tools which target either parasites or vectors. Much effort has been made to improve the understanding of the biology and ecology of the *Anopheles* mosquito in general and in particular members of the *Anopheles gambiae* (*sensu* *lato*) complex which are responsible for the majority of malaria transmission in sub-Saharan Africa. Vector control is one of the major components of the malaria control strategy in many endemic countries and its efficacy in reducing the malaria burden has been widely demonstrated [2, 3]. For example, between 2000 and 2015 there was a 41% decline in the number of malaria cases and 62% in the number of deaths due to malaria, with an estimated 6.8 million of malaria deaths averted [1, 4]. The standard vector control measures (insecticide-treated mosquito nets and indoor residual spraying) brought a substantial contribution to this effect with, for example, at least 50% of the decline attributed to insecticide treated mosquito nets alone [1, 4, 5].

The spread of insecticide resistance now presents a challenge which has encouraged the development of new tools including genetic control by release of transgenic [6, 7], *Wolbachia*-infected [8] and radio-sterilized [9, 10] mosquitoes. Transgenesis is used to introduce an exogenous gene (transgene) into a living organism so that the organism will exhibit a new property and transmit that property to its offspring [11]. In vector control, the transgenesis approach usually aims to reduce vector competence by blocking pathogen development [6, 12]. Novel approaches for self-sustaining population suppression by reducing female fertility [13] or by biasing the sex-ratio toward males [14] are also being developed.

All of these applications of genetic control anticipate the release of male mosquitoes. Sterile insect technique (SIT) is the most advanced method so far, and consists of releasing large numbers of male mosquitoes into a population to mate with wild females, leading them to lay eggs which do not develop further. Critical to the success of this technique is the ability to attain a favourably effective ratio of mating by released sterile male mosquitoes compared with wild ones [15, 16]. This can be achieved by releasing large numbers of males and ensuring that they have high mating competitiveness [5, 16]. High effective mating ratios are also important for self-sustaining population suppression approaches. High male mating competiveness can facilitate the successful introduction of gene constructs into wild populations by decreasing the number of released males required thereby improving the cost effectiveness of novel self-sustaining interventions [5, 15]. In the Sudanian and Sahelian areas, *An. gambiae *(*s.l*.) populations undergo large seasonal changes in abundance and are greatly reduced or disappear completely during the dry season [17–19]. Consequently, male mosquito releases should take place when environmental conditions maximize survival but at a time when target populations are comparatively low, such as the start of the rainy season. Clearly, male mosquito release control programs can greatly benefit from an improved understanding of male mosquito biology, including factors influencing their survival, dispersal and mating behaviour. They also require knowledge about the size fluctuations of target mosquito populations [20–22].

Mosquito population size is a fundamental parameter that is difficult to estimate accurately. A number of data types can be used to estimate population size, including mark-release-recapture (MRR) data [22, 23], genetic data to estimate effective population size [24, 25] and spatially replicated data [26]. The most common technique to date, MRR, is the most direct method [27]. The simplest estimator of population size is the Lincoln index, which is based on the assumption that the ratio of recaptured individuals to the total captures is equivalent to the ratio of marked individuals to the total size of the population [22]. In the past few decades, many refinements to the Lincoln index have been developed to relax this simple assumption, and to allow additional bionomic parameters (in particular, survival and movement) to be inferred from MRR data [28, 29]. Perhaps the earliest and simplest refinement was made by Fisher & Ford [30], who incorporated a survival parameter to account for the mortality of marked individuals during the recapture period*.* In contrast with the Lincoln index, which assumes no mortality, the Fisher-Ford model gives more reliable estimates of population size if mortality is significant during the study period (as it is likely to be with mosquitoes), and the estimated survival parameter is of additional interest in itself. Many of the further refinements to the Lincoln index have focused on accounting for factors such as age and weather-dependent mortality, and spatial variation in population density [28]. Thus, there now exists a wide spectrum of analytical tools for the study of MRR data, yet the appropriate approach for a given dataset will depend on the characteristics of the data.

The accuracy of estimates obtained by MRR experiments are also influenced by two key parameters, mosquito survival and dispersal. Low survival of marked mosquitoes reduces the statistical power of estimating population size (leading to larger confidence intervals). These parameters can be affected by environmental conditions as well as human activities and behaviour such as human population density, the surrounding habitats, local waste management and hygiene practices, or the use of mosquito control tools [31]. Survival in wild mosquito populations can also be influenced by other ecological effects such as parasite infection [32, 33] or intraspecific and interspecific competition during the larval stage [34]. Mosquito movement is fundamentally linked to their biology: seeking a mate, blood meals, oviposition or resting sites [35, 36]. Additionally, released individuals may sometimes suffer from decreased survival due to carry-over effects of laboratory rearing conditions [37, 38] and the negative effects of handling, marking dyes [39] and the release procedures [27]. The overwhelming majority of past MRR studies have focused primarily on females, as they are directly involved in pathogen transmission [27], and are easier to recapture in number inside human dwellings. There is therefore a strong need for estimates of population size, survival and movement based on male MRR experiments that are more pertinent to vector control approaches based on male releases.

The main objective of this study was to determine, *via* repeated MRR experiments, the seasonal variation in population size and to estimate the survival and dispersal of *Anopheles coluzzii* males in a small sub-Saharan Africa village. We estimated population size and daily survival by applying Bayesian inference to a probabilistic version of the Fisher-Ford model. The low number of parameters in the underlying model (survival and population size in each study period) facilitated their estimation from the data, and the Bayesian inference allowed us to evaluate the credible intervals and thus our confidence in these estimates. Net dispersal distance (straight-line distance between the release and recapture locations for each recaptured mosquito) was measured for males released in the centre, edge and outside the focal village. Finally, the effect of the source of the released mosquitoes was assessed by comparing the parameters obtained from field-collected mosquitoes with those of a well-established insectary colony. This study estimates the size of target populations using male-based MRR experiments and the dispersal and survival of released males, which are key parameters for the implementation of vector control programs based on male release strategies.

## Methods

### Study site

The survey was conducted in the village of Bana, in the western Burkina Faso humid savannah. Situated 20 km west of Bobo-Dioulasso (12°36′00″N, 3°28′59″W), the village has two main inhabited areas, separated by a small river: Bana Village and Bana Market. Bana Village is the principal area and includes the village’s administration. Bana Market is the economic centre of the village and holds a weekly seasonal market which attracts many people from surrounding villages. Bana Village is a cluster of about 65 compounds with about 380 inhabitants (local census, October 2014**)**. Each compound is a family unit consisting of between two and ten houses, mostly mud-built. The main activities in the village are arable subsistence farming and stock farming.

This region is characterized by two seasons: a wet season from June to September and a dry season from November to April, with October and May being transition months. The mean annual rainfall in the village is about 800 mm (maximum in September, minimum in January) with a mean temperature of about 27 °C (22 °C monthly mean minimum and 32 °C monthly mean maximum).

Malaria is endemic in this region. Nevertheless, the national vector control program is reasonably well implemented with very high percentage of mosquito bednet coverage (around 98%, even though most of these are time old mosquito bednets). Three members of the *Anopheles gambiae* (*s.l.*) species complex are present in the study area: *An. coluzzii*, *An. gambiae* and *An. arabiensis. Anopheles coluzzii* is the dominant malaria vector in Bana, while *An. gambiae* and *An. arabiensis* are occasionally found, mainly in wet season samples. The highest mosquito density is reached around September-October and the lowest around January-February. *Anopheles nili* and *An. funestus* are also occasionally found in low numbers (< 1%).

Four MRR experiments were carried out in Bana village during two consecutive years; two at the end of the wet season at peak mosquito density (September 2013 and October 2014) and two at the end of the dry season when mosquito densities are low (April 2014 and May 2015). Each MRR experiment used the protocol described below.

### Pre-release phase: Production, sexing and marking of mosquitoes

For each experiment around 5000 (3407–5273) adult male mosquitoes were released. In the first experiment the mosquitoes used were collected as larvae from the field and reared to adults (field-collected, immature-sourced *An. gambiae* (*s.l.*) mosquitoes) under insectary conditions (similar to the protocol used for rearing the insectary colony larvae, described below). Due to limited availability of larvae in the field, especially during dry seasons, subsequent experiments used *An. coluzzii* males from an insectary colony of the IRSS (Institut de Recherche en Sciences de la Santé, Bobo-Dioulasso, Burkina Faso) (Table 1). This strain was colonized in August 2008 (and refreshed in 2012) from gravid female adults collected in Village 7 of the Kou valley (VK7) in western Burkina Faso.

**Table 1:** Number of mosquitoes released by location for the sequential mark-release-recapture (MRR) experiments in Bana village, Burkina Faso

Experiment	MRR1 *An. gambiae* (*s.l.*)^a^	MRR2 *An. coluzzii* ^b^	MRR3 *An. coluzzii* ^b^	MRR4 *An. coluzzii* ^b^
	September 2013	April 2014	October 2014	May 2015
Centre of the village	1146	1878	1665	1807
Edge of the village	1103	1734	1684	1653
Outside the village	1158	1655	1673	1813
Total number released	3407	5267	5022	5273

^a^ *An. gambiae* (*s.l*.) is for a range of wild *Anopheles gambiae* complex mosquitoes caught in an area where *An. coluzzii* is known to be highly predominant

^b^ *An. coluzzii* is for a insectary-sourced colony

The strain was reared in a climate-controlled room maintained at a temperature of 27 ± 1 °C and 70 ± 10% relative humidity. The light regime was of LD 12/12 h photoperiod, including dusk (1 h) and dawn (1 h). Females were allowed to oviposit in plastic Petri dishes containing a wet sponge covered by filter paper. Eggs were collected and hatched in plastic trays (about 30 cm diameter) containing spring water (1 l per tray). Larvae were reared (*c.*200–250 larvae per tray) and fed with the fish food Tetramin®baby (Melle, Germany). Pupae were collected and placed in small plastic cups inside a fresh 30 × 30 × 30 cm insect cages (produced locally) for emergence. The cages were labelled with the name of the strain and the emergence date, and provided with 5% (*w*/*v*) glucose solution.

The first day after emergence, males were separated from females and placed in adult cages for marking. Mosquitoes of the same age were allocated into the same cage at the maximum of 700 mosquitoes per cage. Depending on the number of mosquitoes that emerged each day, male mosquitoes aged from 2 to 6 days were used. During the marking process, a similar number of mosquitoes from each age group were allocated sequentially into three groups in decreasing age order until there were approximately 1500–2000 mosquitoes per group. This method gave a similar mean age of mosquitoes in each group. Each group was marked with a different colour dye: red, blue and green, respectively, corresponding to different release points. The number of males mosquitoes marked with each dye was carefully recorded. The dye used for marking was the Bioquip® colour powder (Bioquip products 2321 Gladwick Street Rancho Dominguez, CA 90220, USA; Ref: 1162 B, R, and Y).

In order to dust the males, a small amount of coloured powder was placed in a 25 ml glass tube. The tube was sealed with cotton wool and then shaken to coat the walls with the dye. A few mosquitoes at a time (10–20) were taken from the cages and transferred to the tube using a mouth aspirator. The tube was shaken slowly for 20–30 s to transfer the dye powder to the mosquitoes. The marked mosquitoes were then transferred to a new cage. The procedure was repeated until the required number of marked mosquitoes was reached. Sugar-water was available ad libitum to all marked mosquitoes. In our experience, this method provides consistent marking, with usually 100% marking success (unpublished data). For each MRR experiment, mosquitoes were marked at least 24 h prior to their release in the field to allow for the removal of dead mosquitoes and those obviously weakened by the marking procedure.

### Release phase

**Fig. 1:**
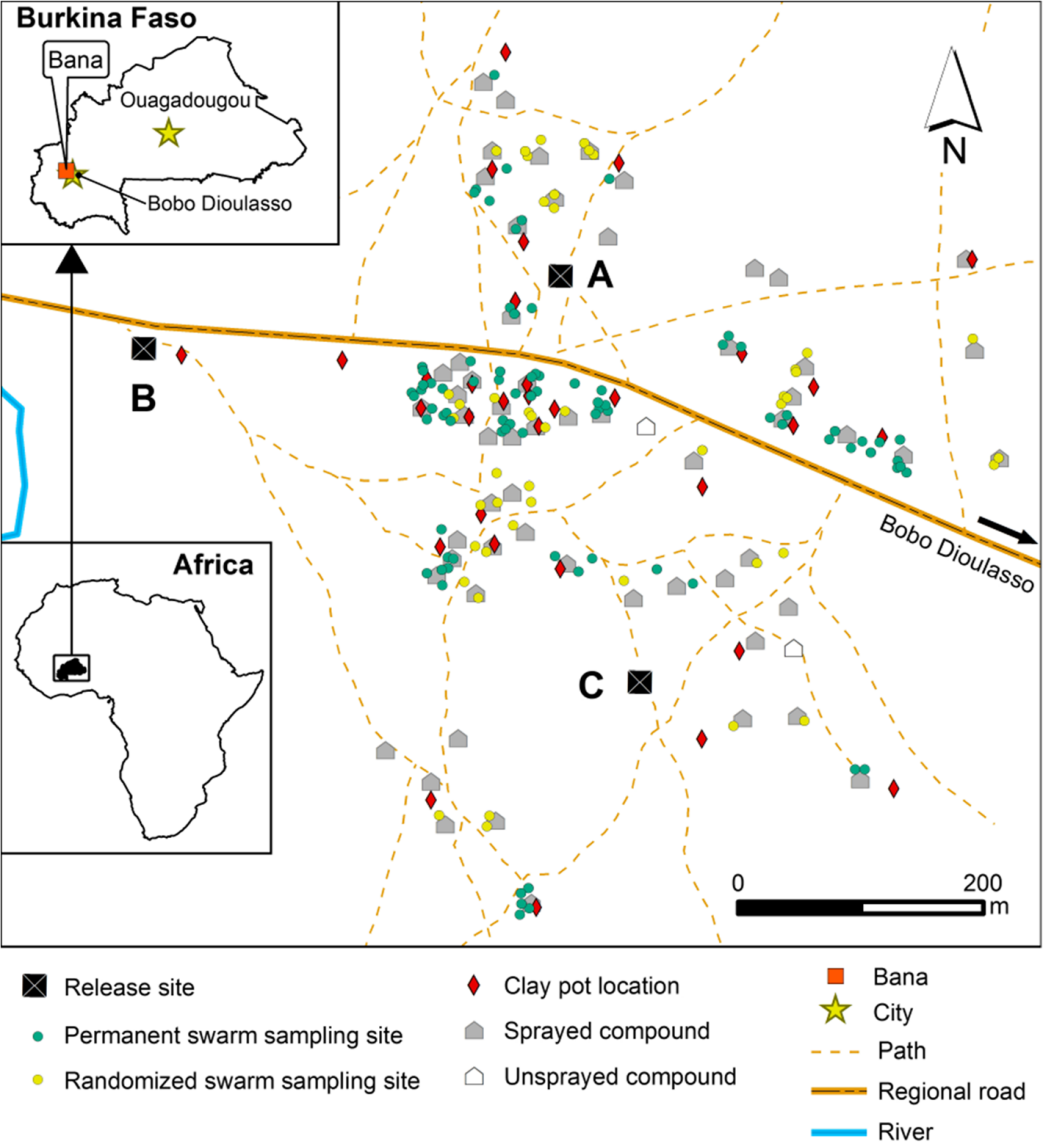
Release and collection sites in Bana village. Permanent swarm sampling sites are where swarms were checked and collected every day; randomized swarm sampling sites are where swarms were checked and collected every three days

In each experiment, marked mosquitoes were released on the same day at around 16:00 (about two hours before swarming) by opening the travel cages and allowing free exodus. Mosquitoes that did not leave were counted and subtracted from the released total. The releases were made in three different locations: in the village centre, at the village edge and at a point 200 m outside the village (Fig. 1). Global positioning system (GPS) coordinates were recorded. A single colour of mosquito was released at a particular release point. The same colours were used at the same release locations for the first three MRR experiments. For the fourth MRR experiment, however, the colours used in the release locations were interchanged in other to observe any potential effects of dye colour on the recapture rate in field conditions, as has been demonstrated on longevity in laboratory conditions [39].

### Recapture phase

Mosquito recaptures took place for 7 days following release (except for the first MRR experiment, when there were only 5 recapture days, due to local conditions on the sixth and seventh days on which field collections were not possible). Three different recapture methods were used: swarm collections, pyrethroid spray catches (PSC) inside houses and clay pots placed throughout the village.

Swarm sampling started on the evening of the release day using a well-established sweep net collection method [40, 41]. As the number and size of swarms varied substantially between seasons, it was not possible to collect a fixed number of males per sample, so a representative sample was collected throughout the village. Previous surveys carried out by our team (monthly swarm collections over one year) had allowed mapping of principal swarm locations and their appearance frequencies. These were divided into 3 groups according to the frequency of swarm appearance in the particular location: high (swarm appearance frequency above 75% in that location); medium (swarm appearance frequency between 25 and 75%) and low (swarm appearance frequency under 25%). The high and medium frequency swarm locations were all checked daily and all the swarms that appeared were collected. The low frequency swarm locations were randomly divided into three subgroups. Each of these subgroups were checked every three days and all swarms found to be present were collected (Fig. 1).

Pyrethroid spray catches started the morning following the release and continued for 7 days. A set of 20 compounds were selected randomly each day. For each compound selected, a single room (sleeping room) was chosen for sampling. Although some compounds were selected more than once during the seven days, a different room (from a different house inside the same compound when applicable) was selected and no room was sampled twice during the survey period.

Clay pots, when suitably humidified, may represent refuges for mosquitoes especially during the dry season [42, 43]. Before each release, 32 clay pots were placed at different randomly-selected locations inside the village (Fig. 1) and GPS coordinates of each were recorded. The precise locations of the clay pots in the area randomly chosen were selected to optimize the clay pot for *Anopheles* mosquito collections (with the main objective being to assess their utility in *Anopheles* mosquito collections). Some of the criteria taken into consideration were accessibility to the team, shade to maintain humidity, reduced access to children or cattle. Clay pots were humidified each evening during the study period and were inspected daily early each morning (6:00 to 7:00 am). All mosquitoes in each clay pot were collected using a mouth aspirator.

The location of each collection was recorded and mapped using a GPS (Garmin GPS, Canton de Schaffhouse, Switzerland) device, series GPSMAP®62.2.3. Mosquitoes were identified morphologically. All *An. gambiae* (*s.l.*) mosquitoes were counted, checked for dust marking and preserved in 80% ethanol.

### Data analysis

For each of the four experiments, we estimated both the size of the wild population and the mortality rate of the released mosquitoes. Recapture proportion, the number of recaptured mosquitoes as a weighted proportion of the number released, between experiments was separately compared using either proportion tests or binomial-family generalized linear models (GLM) with stepwise factor level reduction testing. Analysis of variance (ANOVA) with stepwise deletion testing was used to assess the influence of season, capture method and release location on the net distance dispersed (the distance between release and recapture points in meters) by the recaptured mosquitoes. The degrees of freedom presented with *F*-values are those associated with the factor of interest and the error/ residual degrees of freedom of the model. Statistical analysis used R 3.3.1 [44].

### Population size estimates

The Fisher-Ford model for estimating population size and survival is based on the following assumptions: (i) the marked individuals die at a constant rate during the study period; (ii) the marked individuals are equally as likely as other individuals in the population to be observed; and (iii) the wild mosquito population is constant over the sampling period. We denote *s* to be the probability that any marked mosquito survives any given day (and remains in the study area), which may or may not differ between the four experiments. According to these assumptions, the probability of recapturing each released mosquito on a given day *d* can be approximated as the proportion of the population that is sampled with a given recapture method on that day. We write this as

1 $$ \frac{m_d}{R{s}^d}=\frac{c_d}{N} $$

where *m*_*d*_ is the number of marked male mosquitoes captured on day *d* from a release of *R* marked mosquitoes on day 0, and *c*_*d*_ is the number of non-marked mosquitoes from the population whose size is *N*. Note that we ignore (i) the mortality of marked mosquitoes caused specifically by their recapture, which is justified for our data because the recapture rates are low; and (ii) the effect of releasing marked males on increasing the population size, which is justified because the population sizes are large compared to the number of males released. Note also that in the limiting case of *s* = 1 (no mortality), Eq. 1 is essentially a simple estimate of population size, with $N=\frac{R{\sum}_d{c}_d}{\sum_d{m}_d}$ which is defined as the Lincoln index [45].

Here we apply the Fisher-Ford model (Eq. 1) [30] in a Bayesian framework to account for sampling variance. The Bayesian approach works on the basis that the actual number of recaptures is a random sample from a distribution defined by the number of released mosquitoes and the recapture probability. Specifically, we suppose *m*_*d*_ is drawn from the binomial distribution defined by *R* trials (released mosquitoes) where each trial has probability ${s}^d\frac{c_d}{N}$ of success (recapture),

2 $$ {m}_d\sim binomial\left(R,{s}^d\frac{c_d}{N}\right). $$

Finally, we approximate the binomial by the simpler Poisson distribution, which is justified by the low recapture rates in our data,

3 $$ {m}_d\sim poisson\left(R{s}^d\frac{c_d}{N}\right). $$

Equation 3 allows us to compute the likelihood of recapturing exactly *m*_*d*_ marked and *c*_*d*_ unmarked mosquitoes for any given values of *R* , *s* and *N*, using the probability mass function of the Poisson distribution,

4 $$ f\left({m}_d,{c}_d|R,N,s\right)=\frac{{\left(\frac{R{s}^d{c}_d}{N}\right)}^{m_d}{e}^{\left(\frac{R{s}^d{c}_d}{N}\right)}}{m_d!}. $$

For each experiment and capture method, we assume conditional independence of recapture numbers on each day of the study, so that the log-likelihood of the recapture sequence (*m* = {*m*_*d*_}_{*d* = 1 .  . *D*}_ , *c* = {*c*_*d*_}_{*d* = 1 .  . *D*}_ where *D* is the number of sampling days in the experiment) is the sum of per day logged likelihood functions,

5 $$ Lf\left(m,c|R,N,s\right)=\sum_{\left\{d={d}_0..D\right\}}\mathit{\log}\left[f\left({m}_d,{c}_d|R,N,s\right)\right], $$

where *d*_0_ is the first day of recapturing after release (*d*_0_ = 0 for swarm recapture data and *d*_0_ = 1 for PSC data). The log-likelihood allows point estimation of *N* and *s* because it is maximal when these parameters are such that the data {*m*_*d*_}_{*d* = 1 .  . *D*}_ most closely follows its expectation $R{\left\{{s}^d\frac{c_d}{N}\right\}}_{\left\{d=1..D\right\}}$. Here, in order to estimate *N* and *s* with associated confidences, we combine Eq. (5) with Bayes theorem to calculate *p*(*N*, *s* ∨ *m*, *R*, *c*), the unnormalised posterior distribution for *N* and *s*, giving.

6 $$ p\left(N,s|m,c,R\right)=Lf\left(m,c|R,N,s\right)\varPsi (N)\varPhi (s), $$

where *Ψ*(*N*) and *Φ*(*s*) represent prior knowledge of population size and survival, respectively. Since we had little prior knowledge of population size and survival before these experiments were carried out, we use relatively diffuse distributions in the analyses that follow (see below). For each of the four MRR experiments, we can use Eq. (5) to obtain one or several estimates of the male population size *N* and marked male survival *s*, provided that sufficient numbers of mosquitoes were recaptured with more than one method (for example PSC and swarm sampling). Alternatively, the data from different capture methods can be combined by multiplying the relevant likelihood functions together.

### Posterior sampling

We used uninformative priors to reflect a lack of prior information on the population size and mortality. More specifically we set

$$ \varPsi (N)= LogNormal\left(13.1,1\right) $$

$$ \varPsi (s)= beta\left(8,3\right). $$

To sample the posterior distributions, we applied a Metropolis-Hastings MCMC algorithm in the software Mathematica (version 11.0) with 2 chains of 10^5^ iterations in each case (discarding the first 9^∗^10^4^ iterations from each chain).

### Comparing the model estimates

We estimated the wild mosquito population size and daily survival rate during the four study periods using two versions of the model described above: a standard version and a constant survival version. The standard version model, which allows a different survival rate for each study period was realized in three sub-versions depending on the data source (swarm sampling, PSC sampling, or swarm and PSC sampling combined together). The constant survival version assumes a fixed rate of survival across all study periods.

The modelled estimates of population size were compared by ANOVA and *post-hoc* Tukey Honest Significant Difference multiple comparison tests using 40 model estimates per condition. The wide variation in population predictions between the MRR experiments led to complex interactions and to clarify the picture, the data were also analysed as a function of Technique (model assumptions and input data) within each MRR.

## Results

### Recapture rates

The four experiments had varying recapture rates (*χ* ^2^ = 62.09, *df* = 3,0, *P* < 0.001) (Table 2). The recapture rates did not differ between the two wet season experiments (*χ* ^2^ = 2.35, *df* = 1,2, *P* > 0.05) suggesting that there is no difference in this measure between mosquitoes from the two sources: one used adults reared from field-collected immature mosquitoes, the other used insectary-sourced adults. Recapture rates in the dry season experiments were both lower (*χ* ^2^ = 13.36, *df* = 1,2, *P* < 0.001) and differed from each other (*χ* ^2^ = 15.18, *df* = 1,2, *P* < 0.001) with the fourth experiment having the lowest recapture proportion (0.4%) (Table 2). The recapture rates of males released in the centre of the village, were higher than those for males released outside and on the edge of village in the three experiments, but not in the fourth (Table 2), highlighting a potential problem with the dust marking for that experiment (see Discussion).

**Table 2:** Summary of recaptures by collection method over the four experiments

	Unmarked	Number of marked recaptures^a^	Marked by method^c^ (%)	Total recaptured^d^ (%)
October 2013 (wet season)				
Swarm	4454	34 (21, 8, 5)^b^	0.76	1.00
PSC	1162	9 (7, 1, 1)	0.77	0.26
Clay pot	227	1 (1, 0, 0)	0.44	0.03
All	5843	44 (29, 9, 6)		1.30
May 2014 (dry season)				
Swarm	211	29 (22, 3, 4)	12.08	0.55
PSC	139	16 (10, 2, 4)	10.32	0.30
Clay pot	13	4 (2, 1, 1)	23.53	0.08
All	363	49 (34, 6, 9)		0.93
September 2014 (wet season)				
Swarm	7641	72 (52, 5, 15)	0.93	1.40
PSC	938	10 (8, 0, 2)	1.05	0.20
Clay pot	235	4 (4, 0, 0)	1.67	0.08
All	8814	86 (64, 5, 17)		1.70
April 2015 (dry season)				
Swarm	1421	18 (1, 3, 14)	1.25	0.30
PSC	228	2 (0, 0, 2)	0.87	0.03
Clay pot	26	2 (0, 0, 2)	7.14	0.03
All	1675	22 (1, 3, 18)		0.36

^a^Marked (No.) is the number of marked mosquitoes recaptured by each method

^b^Numbers in parentheses are recaptures from release points: A (centre of the village), B (outside the village) and C (edge of the village), respectively

^c^‘Marked by method’ is the percentage of marked mosquitoes in the catch of each collection method (irrespective of dye colour)

^d^Total recaptured is the percentage of marked mosquitoes recaptured by each collection method from all released (irrespective of dye colour)

As expected, there was variation in the proportion of recaptures according to the collection method used (*χ* ^2^ = 255.94, *df* = 2, *P* < 0.001). Swarms (male-oriented collection method) caught consistently more males than the other two methods, with 76% of all marked males recaptured this way across the experiments. Clay pots performed the worst (only 11 marked males were found in them over the four experiments) and their data was not used in the modelling.

Even though the swarm collections recaptured more marked mosquitoes than PSC in absolute number, the two methods did not differ significantly from each other in the likelihood of recapture (number of marked mosquitoes recaptured by a specific method as a proportion of the total nu4mber of mosquitoes collected with this method) (for MRR1: *χ* ^2^ < 0.01, *df* = 1, *P* = 1; for MRR2: *χ* ^2^ = 0.14, *df* = 1, *P* = 0.71; for MRR3: *χ* ^2^ = 0.04, *df* = 1, *P* = 0.85; for MRR4: *χ* ^2^ = 0.3, *df* = 1, *P* = 0.86).

### Population size

**Fig. 2:**
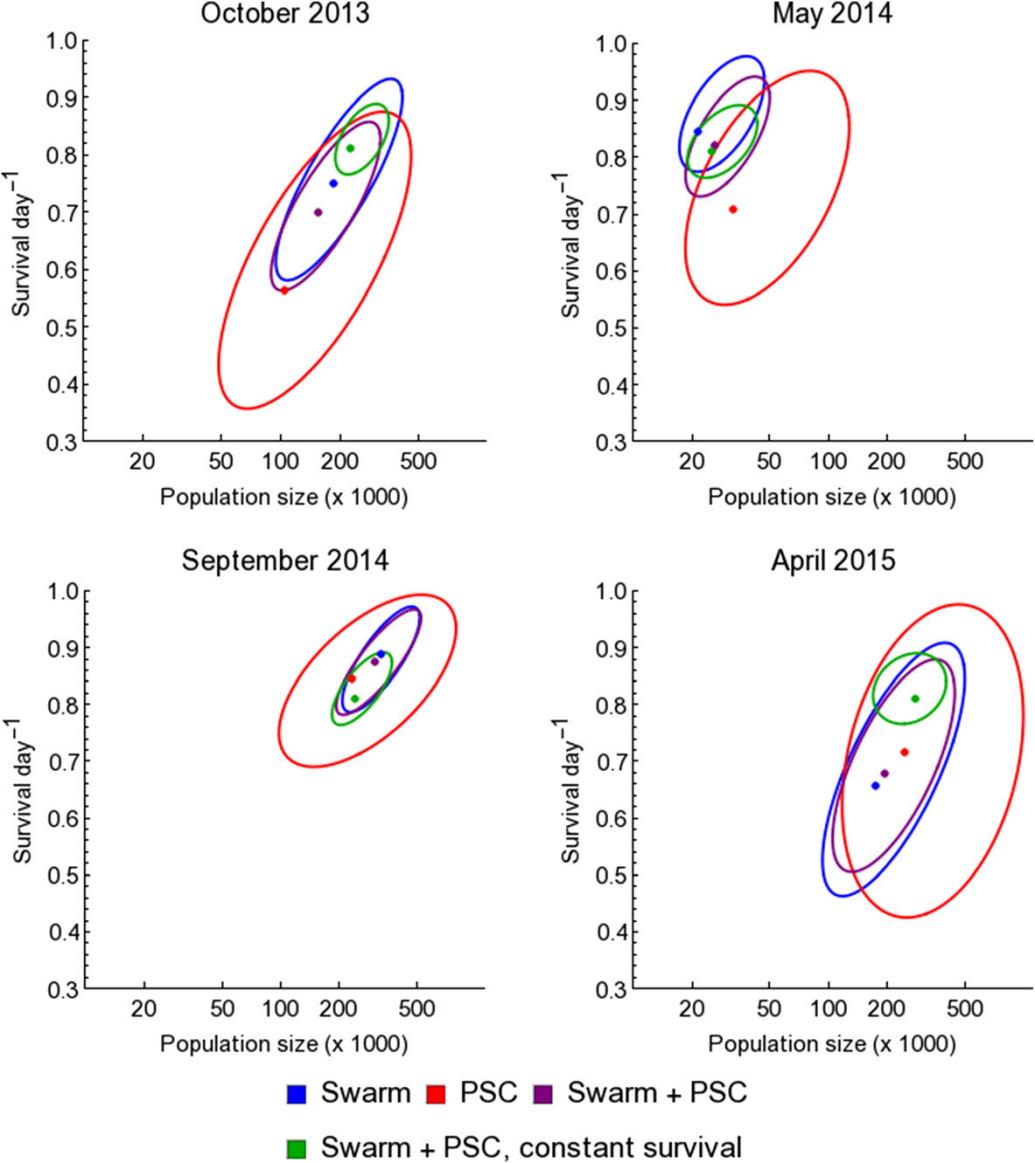
Estimated size of the background population and daily survival during the four study periods. For each period, the ellipses demarcate the most probable parameter combinations according to the corresponding data and model; in each case the ellipse contains 95% of the posterior density. The corresponding dots plot the maximum a posteriori probability (MAP) estimates. The standard model (*blue*, *red* or *purple* depending on the data source) allows a different survival rate in each study period, while the “constant survival” model (*green*) assumes a fixed rate of survival across all study periods

Estimates of the male population size from the different models described above were largely consistent (Fig. 2). Across all models and collection methods, we estimated the male population to be in the range of 100,000–500,000 in the wet season. The May 2014 experiment indicated a population in the range 10,000–50,000 suggesting a decline by an order of magnitude during the dry season. We did not see this in the following dry season (April 2015), but this estimate may be influenced by comparatively poor recapture rate in this experiment (2.5 times lower than in May 2014).

In all MRR experiments there was variation in estimated population size between the models (*F*_(3, 156)_ = 5.76, *P* < 0.001). The estimates arising from PSC data had greater range than those of the swarm data, but the effect of method on the predictions was not consistent between MRR experiments. In the first wet season experiment, swarm samples led to higher population estimates than PSC samples (*P* < 0.003), in the second wet season experiment the methods made similar predictions (*P* = 0.11). In both dry seasons, the swarm samples led to lower predictions than PSC (*P* < 0.001 in both). Using data from both methods gave greater certainty to the estimates (reduced the variance surrounding the estimates) as did constraining mortality to a value estimated across all experiments (constant survival model version). When combining the PSC and swarm data to predict the population sizes, using a single fixed survival rate derived from all experiments led to higher population estimates only in the first MRR experiment characterized by the highest recapture rates (*P* < 0.001). In the other three experiments the effect of doing this was not significant (*P* > 0.15 in all cases).

### Survival of released mosquitoes

**Fig. 3:**
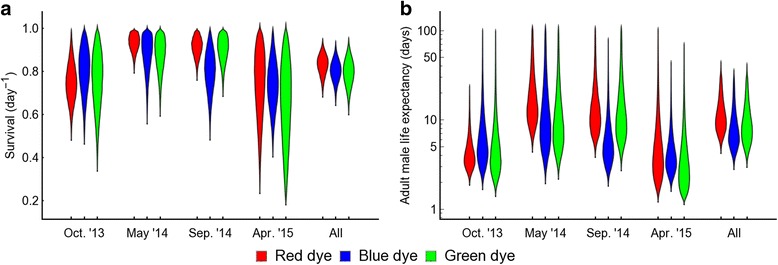
Effects of colour dye on survival and life-history estimates. For each period, the bands plot the posterior distributions of **(a)** survival s and **(b)** life-expectancy calculated as (1–s)^-1^ where s has been estimated from both swarm and PSC data arising from the given colour dye from that period. For the first three experiments, the colours are confounded with their corresponding release locations (*red* dye, centre of village; green dye, edge of the village; and *blue* dye, outside village). The rightmost band plots the posterior distributions when all these data are combined and it is assumed that there is a fixed rate of mortality across all study periods. Oct. ‘13, May ‘14, Sep. ‘14 and Apr. ‘15 indicate the four study periods: October 2013, May 2014, September 2014 and April 2015, respectively

**Fig. 4:**
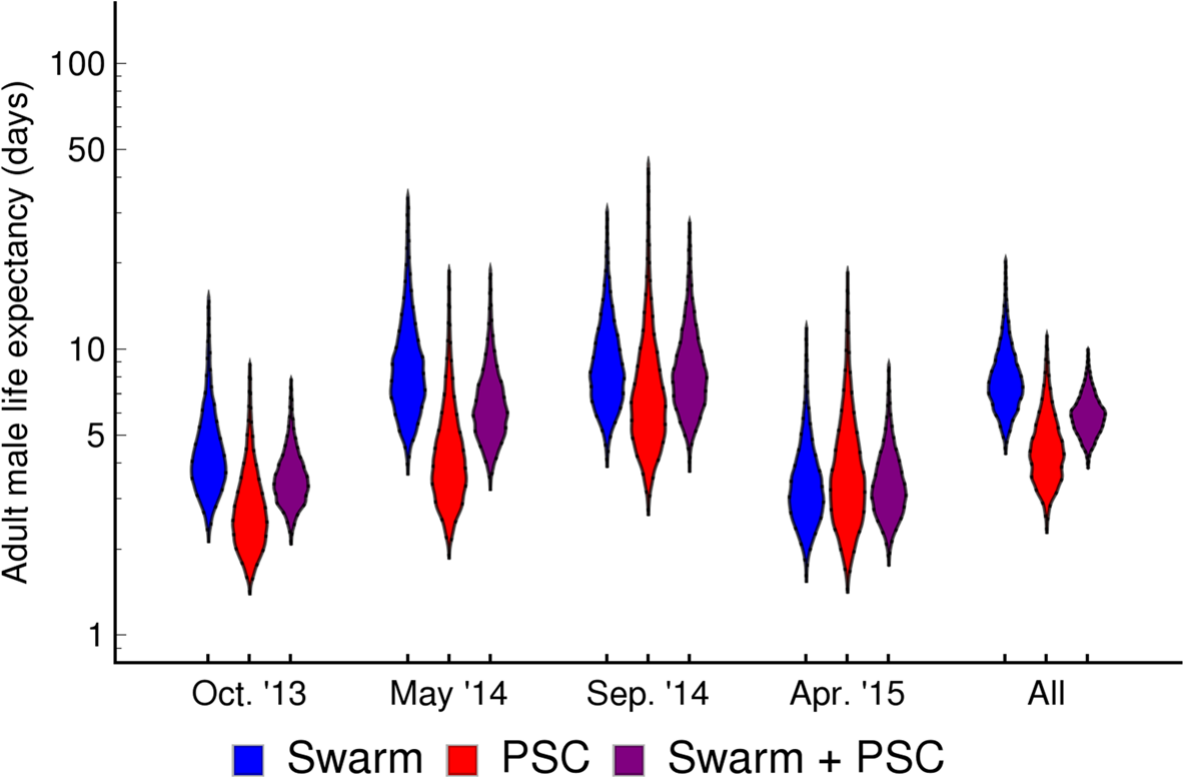
Estimated life-expectancy of the marked mosquitoes during the four study periods. For each period, the bands plot the posterior distributions of life expectancy calculated as (1–s)^-1^ where s is the posterior distribution of daily survival, using either swarm, PSC, or both data from that period. The rightmost band plots the posterior distributions when all these data are combined and it is assumed that there is a fixed rate of mortality across all study periods (“constant survival” model of Fig. 2). Oct. ‘13, May ‘14, Sep. ‘14 and Apr. ‘15 indicate the four study periods: October 2013, May 2014, September 2014 and April 2015, respectively

The Fisher-Ford model was also used to estimate the survival of the released mosquitoes (Figs. 2, 3a, Table 3), essentially from the decline in the day-by-day recapture rates. Using the individual experiments gave rise to wide credible intervals due to high variation in day by day recapture. Substantial variation was also observed between the different experiments. There was not an identifiable seasonal pattern in survival rates, suggesting that factors other than weather may play an important role in mosquito survival in the field. Estimation of survival rates allows estimates of life expectancy to be calculated (Fig. 4). Life-expectancy estimates shown in Fig. 4 rely on the same assumptions used to estimate survival; in particular that mosquito survival is constant.

**Table 3:** The modelled mean estimates of survival (s), and their standard deviations (SD), based on recaptures by two different methods and when the data from both were combined

Experiment	October 2013		May 2014		September 2014		April 2015		All data	
	s	SD	s	SD	s	SD	s	SD	s	SD
Swarm	0.739	0.063	0.864	0.040	0.878	0.044	0.674	0.097	0.789	0.107
PSC	0.615	0.129	0.738	0.066	0.837	0.053	0.701	0.104	0.723	0.122
Both^a^	0.700	0.068	0.835	0.041	0.865	0.029	0.692	0.064	0.773	0.094

^a^Estimates with data from swarm and PSC combined together

Apart from the atypically low-recapture experiment in April 2015, the survival (Figs. 2, 3a, Table 3) and life expectancy (Figs. 3b, 4) estimates derived from PSC were both lower and less certain than those which used the swarm capture data (*P* = 0.41 for April 2015, all other *P* < 0.001). The greater numbers of mosquitoes caught in swarm delivers narrower estimates than with PSC and combining the data from both methods allows further narrowing. Combining the data from both methods may be the most representative of overall male survival as it includes data from a greater range of male behaviour. Combining the data across all experiments to give a single measure of life expectancy gives narrower intervals but should be viewed with caution due to the assumption of this model that survival rates are invariant through time.

### Dispersal

**Fig. 5:**
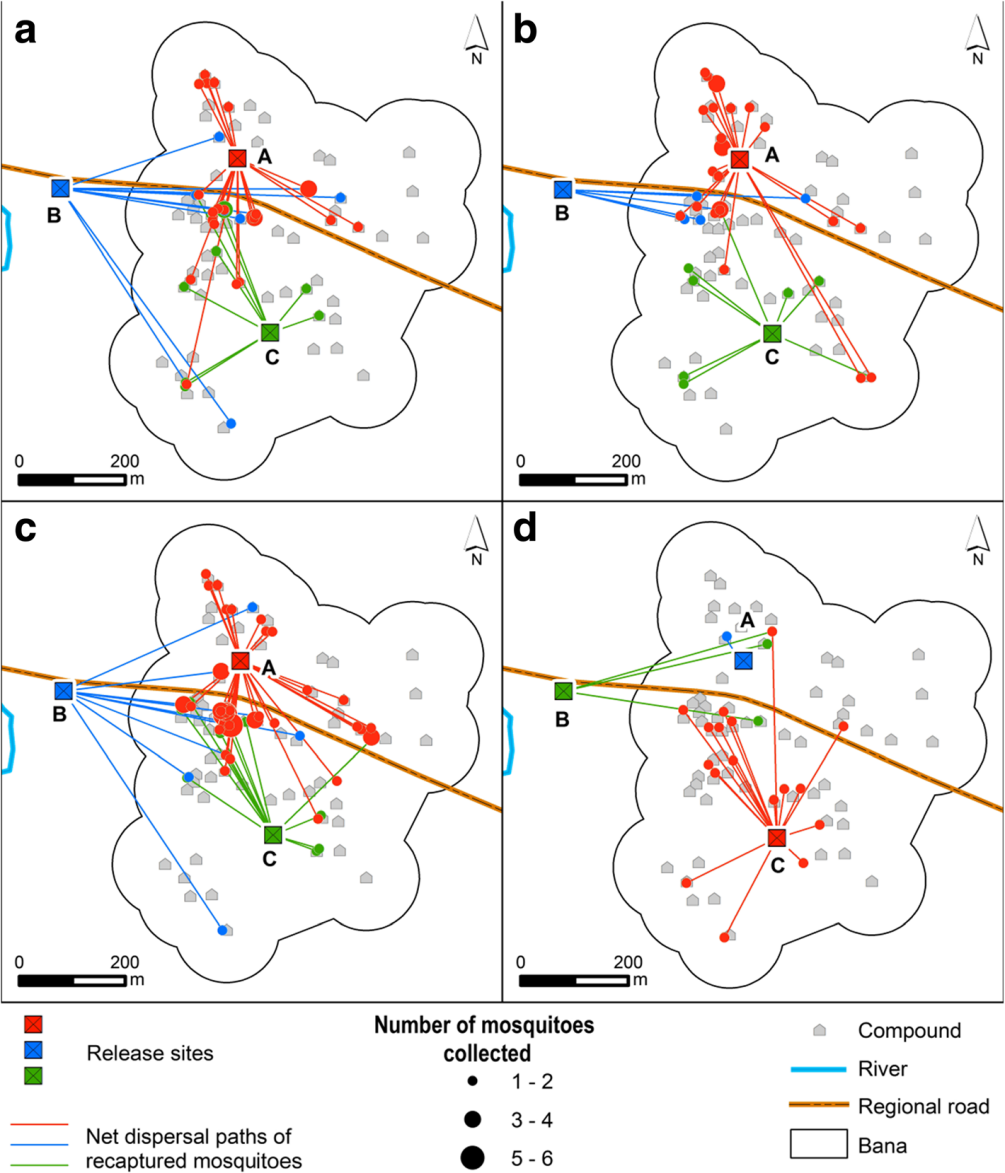
Spatial distribution of recaptured mosquitoes during the four experiments: October 2013 (**a**); May 2014 (**b**); September 2014 (**c**); and April 2015 (**d**). *Red*, *blue* and *green* lines indicate the net dispersal paths of red-coloured, blue-coloured and green-coloured released mosquitoes, respectively. A, B and C are the release locations, the village centre, outside the village and the edge of the village, respectively

**Fig. 6:**
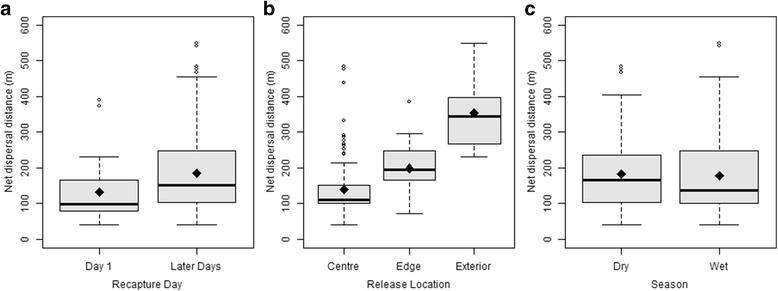
Net dispersal distances of released mosquitoes. The net dispersal distance as a function of time elapsed since release (**a**), release location (**b**) and season (**c**). The bold black line is the median distance observed. The bottom and top of the box show the first and third quartiles, respectively, the black diamond indicates the mean value. The vertical whisker lines indicate 1.5 times the interquartile range of the data beyond which the ‘outliers’ are illustrated as individual dots

The ability to find mates constitutes an important element of mosquito fitness, and mobility is key to this. Measurements of net dispersal distance allowed us to assess some of the factors that may be important to mobility, whilst the spatial distribution of recaptures gives an indication of “mosquito preferred” areas within the study village (Fig. 5). Overall, the mean net distance travelled by male mosquitoes between release and recapture ranged from a minimum of 40 m to a maximum of 549 m and did not vary between experiments (*F*_(3, 192)_ = 0.13, *P* ˃ 0.05), which means no variation between the two seasons of the year (Fig. 6c), nor between the three recapture methods used (*F*_(2, 195)_ = 0.18, *P* ˃ 0.05) but there was a significant effect of recapture day (time elapsed in days between the day of release and the day of recapture). On the first day of recapture, mosquitoes were found closer to their release point than on any subsequent day (*F*_(1, 197)_ = 12.77, *P* < 0.001), though all subsequent days were indistinguishable (*F*_(6, 191)_ = 0.61, *P* > 0.05) (Fig. 6a). The strongest effect on mosquito dispersal was where they were released (*F*_(2, 197)_ = 83.52, *P* < 0.001) which explained more than 44% of the variance in the data (Fig. 6b). The mosquitoes released outside the village moved the furthest, followed by the ones released at the edge of the village. The mosquitoes released at the centre of the village, though recaptured in the greatest numbers, showed the least dispersal.

## Discussion

A good understanding of male mosquito biology, behaviour and dynamics is of crucial importance for being able to implement male mating-based control methods such as SIT or genetic control methods. Despite increasing interest from the research community, knowledge of male mosquito biology is still insufficient for these to be used with efficiency as targets for vector control [5]. In this study, we used four rounds of MRR experiment in the same village both to improve our understanding of male mosquito bio-ecology in this ecotype and to assess the potential of a well-established male *An. coluzzii* colony to be used as a tool to estimate numbers and behaviour in a wild population.

In this part of Africa, swarms are the primary arena for mate-finding in *An. coluzzii* [46, 47]. Though some mating has been noted in houses in other contexts [48], the proportional contribution of this remains poorly estimated. In this experiment 24% of recaptured males were found indoors, but this does not necessarily indicate whether interior mating is occurring and may simply indicate resting preference. The swarm sampling method caught the highest absolute numbers (even though the probability of a male mosquito being collected in each method was similar) and thus gave narrower credible intervals, but including the data from PSC narrowed those intervals further and allowed another aspect of male biology to contribute to the estimates made. Independently, the estimates of survival and wild population size derived from PSC and swarm captures were broadly similar. The contribution to recapture of the clay pots was not rewarding for the effort made in placing the pots and in checking them. With so few marked males found this way, it is not a method we would recommend for future MRR studies in this location.

In this study, males sourced from an insectary colony and those field-collected as immature sourced mosquitoes were both found to participate in swarms at similar rates (a similar proportion of released individuals were observed in swarms). In fact, the proportions of released mosquitoes recaptured in swarms to the total number of mosquitoes caught in swarm in both cases (Table 2), were not significantly different. This observation is supported by the long-established laboratory ‘G3’ *Anopheles* line still displaying swarming in laboratory conditions when offered suitable cues [49]. Nevertheless, this swarming should be considered with some caution as it is known that rearing conditions in insectaries affect some of the other important factors that play roles in *Anopheles* mating competitiveness [50, 51]. In addition, a number of previous observations of aspects of the fitness of laboratory-sourced male mosquitoes indicate a loss of performance compared to the wild counterpart [49, 52, 53]. This colony was refreshed by the addition of wild stock in 2012 and this could have contributed to the maintenance of natural behaviour. It should be noted though, that this evidence of swarm participation does not assess their ability to mate competitively.

As with the estimates of population size, the estimates of survival made by using only the swarm sampling data were not dissimilar to those of the PSC, although the greater numbers from swarms led to smaller credible intervals. The PSC data does give added value in including another aspect of male behaviour, indoor resting, though general opinion is that male *An. coluzzii* are usually found outdoors as their resting, feeding and mating places are exterior. More can be found indoors when the weather conditions are unsuitable [54].

The consistency of the model estimates between the wet-season MRR experiments is reassuring, but those of the dry-season MRRs contrast substantially. It is unlikely that the population in the dry season (April 2015) is at the same level as that of the wet season when all baseline data and the previous dry season MRR suggest a 10-fold reduction at this time of year. The observed difference between the two dry season experiments may be associated with different effects on mosquito performance of the different colour dyes [39]. For the first three experiments colour was associated with a particular release point, whereas, for the last experiment (April 2015) we varied the colour that was associated with each release point. We found no impact of dye colour on mosquito survival (Fig. 3), nevertheless, since dye colours and release sites are confounded in a large part of this study, it is not really possible to clearly differentiate the effect of each factor without further experiment.

Very few MRR experiments involving male mosquitoes of *Anopheles gambiae* have been published and these few differ markedly in experimental design [27]. Nevertheless, a rough comparison shows that the results of survival and population size estimates with our model are consistent with those of similar MRR experiments. One of the most comparable experiments (swarm sampling, sahelian climate, and release of insectary-reared mosquitoes) is that of Ageep et al. [55], which took place in Sudan with irradiated insectary-reared *An. arabiensis*. Daily survival was estimated at 0.73 in this study, which is within the range of our estimates (Fig. 2, Table 3). In a Burkina Faso village similar to our study site, Costantini et al. [56] estimated the survival and density of *An. gambiae* (*s.l.*) females through MRR experiments conducted over two years. Depending on the year and the statistical method used, this study estimated daily survival rates to be in the range of 0.67–0.95 [56]. The same study reported estimates of mosquito population density ranging between 150,000–350,000, which is similar to the range of our wet season estimates (100,000–500,000) [56]. A number of studies focusing on female *An. gambiae* (*s.l.*) were also carried out in Kenya and Mali and, despite being based on very different study designs, reported survival rates around 0.95, thus broadly consistent with our estimates for male survival [57, 58]. These similarities suggest that the survival rates of male and female *An. gambiae* (*s.l.*) may not be substantially different. In the Malian villages, the estimates of female *An. gambiae* (*s.l.*) population size were around 9000–28,000 depending on the season [57]. These numbers suggest lower population sizes in that region, although they are not dissimilar to our more reliable May 2014 dry season estimates (16,000–53,000).

In this study, several mosquitoes were found to have dispersed over 500 m in net distance from their release point. As the net dispersal distance is a straight-line measure, it is likely to be an under-estimate of the actual maximum flight range of the mosquito. The dispersal distances were not influenced by season (Fig. 6c), suggesting that, as observed for survival, this parameter may broadly remain similar throughout the year in the same locality. In this study, local dispersal was affected by the release location, with mosquitoes released outside the village being recaptured the furthest away. It is possible though, that this is an artefact of the sampling regime as this release site was, on average, furthest from the recapture locations. All recaptures used in the modelling were made within the village as this is where compounds are and where the previous extensive mapping identified swarms locations. Nevertheless, the results suggest that the released mosquitoes tend to disperse toward the core of the village, which also has a higher density of human population (Fig. 5). This lends some support to the hypothesis that *Anopheles* mosquitoes are attracted to human populations. This agrees with other studies of *An. coluzzii* male behaviour which show that their common living places are usually closely associated with human habitats [59], as their movements are mainly associated with feeding and mate seeking. Further studies of male mosquito spatial dynamics (especially those involving an intensive mosquito sampling outside or around village) will be of great help in giving a better understanding of the factors influencing male mosquito dispersal.

The Fisher-Ford model used to estimate population size and survival in these experiments is one of the most commonly used amongst mosquito MRR studies [60]. Our decision to use it here reflects a judgment that this is the simplest possible model that allows our primary parameter of interest (population size) to be estimated without major bias due to the mortality of marked mosquitoes. Although a more complex model would in principle allow investigation of additional biological factors (for example age-dependent survival), the data is not sufficient to readily estimate such factors. The simplicity of the Fisher-Ford model allows greater precision in estimating population size and the Bayesian inference allows the uncertainty in these estimates to be honestly evaluated.

There is some evidence that mosquito daily survival reduces with age in laboratory conditions, due to senescence [32, 61]. There is less evidence that senescence is significant in natural populations where survival rates are generally lower [62]. We thus feel the assumption of constant survival is justified in this study, yet it is worth speculating how it affects our estimates. If adult mosquito daily survival is in fact reducing with age (because of senescence), our estimates of survival will be downwardly biased for young and upwardly biased for old adult mosquitoes. These biases will have little impact on our estimates of population size, which are more sensitive to average daily survival over the study period. If age-dependent mortality is significant, however, the credible intervals that correspond to our estimates of population size will be understated. Moreover, our estimates of longevity may be somewhat inflated, because the constant survival model overestimates the abundance of old mosquitoes in comparison with models that allow senescence. Note that we have estimated survival and longevity for marked mosquitoes, which are likely to be less suited to the field conditions than the native population (due to lab handling, lab adaptation, and effects of the colour marks). We thus expect our estimates of survival and longevity to be conservative with respect to survival and longevity of the native population, irrespective of age-dependency. We have also assumed that the native population is constant in size during each of the four experiments. If the population was in fact growing or shrinking during a particular experiment, then we have essentially estimated the average population size over the duration of the experiment.

One of the strengths of this study was to assess the consistency of our model predictions at the same site during both same and different seasons of the year. As well as the within-experiment variation in recapture rates mentioned above, low recapture rates generally have been a concern during this study. The recapture rates were generally lower than those observed in other MRR experiments using *An. gambiae* (*s.l.*) mosquitoes [27, 55], though these have mostly used females. Nevertheless, these results also showed that repeating the same protocol in the same location can provide replication and compensate for the difficulties of drawing conclusions from a technique with inherently low recapture rates.

## Conclusion

The male mating-based control methods such as the sterile insect technique or other genetic control methods are some of the most promising currently proposed to contribute to ongoing mosquito control in an era of increased insecticide resistance. These will need to be informed by a better understanding of mosquito biology, ecology and behaviour than we have now. This study clearly shows that male-based MRR experiments can be used to estimate some parameters of wild male populations such as population size, dispersal, survival, and an idea of the spatial and temporal dynamics in a given locality. Swarm sampling appears to be the most reliable method for monitoring of male mosquitoes in the field, but should be associated with PSC to have more accurate estimates of population parameters and to capture the breadth of mosquito behaviour. MRR experiments will also be important for investigations and assessment of the field fitness of potential candidate male mosquitoes in future vector control research programs.

## Abbreviations

ANOVA: Analysis of variance
df: Degrees of freedom
GLM: Generalized linear models
GPS: Global positioning system
IRSS: Institut de Recherche en Sciences de la Santé
LD: Light/darkness ratio
MRR: Mark-release-recapture
PSC: Pyrethroid spray catch
SIT: Sterile insect technique
